# Listening in the bog: II. Neural correlates for acoustic interactions and spacing between *Sphagniana sphagnorum* males

**DOI:** 10.1007/s00359-018-1251-7

**Published:** 2018-02-19

**Authors:** Konstantinos Kostarakos, Heiner Römer

**Affiliations:** Institute of Zoology, University of Graz, Universitaetsplatz 2, 8010 Graz, Austria

**Keywords:** Insects, Auditory processing, Distance estimation, Frequency modulation, Signal detection

## Abstract

Males of the katydid *Sphagniana sphagnorum* maintain inter-male distances from one another using agonistic song interactions with a frequency-modulated song that consists of alternating audio and ultrasonic parts. We studied the neuronal representation of this song in auditory receptors and interneurons of receivers, using playbacks of songs that mimicked the absolute and relative sound pressure levels of the two song modes varying with distance. The tuning and sensitivity of both receptors and interneurons strongly determine their responses to the two song modes at different distances. Low-frequency interneurons respond preferentially to the audio mode of the song at larger distances. High-frequency (HF) interneurons respond preferentially to the HF component of the song at close range. ‘Switch interneurons’ are sensitive to both spectral song components, but exhibit a typical activity switch towards the high-frequency mode at distances nearer than 3–6 m. The activity of the latter two groups of interneurons correlates with the distance in the field at which males begin to interact acoustically with their neighbours. Important information about the rate of changes in the song mode is represented by the afferent activity despite the influence of the masking song produced by a sympatric katydid species.

## Introduction

Many species of orthopteran insects form aggregations of singing males (Gerhardt and Huber 2002; Greenfield 2002). The results of analysis of the distances between neighbours have indicated that individuals are rarely close enough to detect each other using any sense other than hearing, and that males within aggregations maintain a given distance from their nearest neighbours (Campbell and Clarke 1971; Meixner and Shaw 1979; Thiele and Bailey 1980; Morris et al. 2018). Such spacing is achieved through acoustic interactions and corresponding movement towards and away from competing males (Bailey and Thiele 1983; Morris 1967; Thiele and Bailey 1980; Meixner and Shaw 1986; Schatral et al. 1984; Chamorro et al. 2007). The ranging hypothesis states that frequency-dependent excess attenuation or degradation of acoustic signals in the time domain are used as sensory cues (McGregor and Krebs 1984; Morton 1986; Naguib and Wiley 2001). However, frequency-dependent excess attenuation appeared to be less relevant for the well-studied Australian katydid *Mygalopsis marki*. Instead, a suprathreshold perceived intensity of 65 dB SPL, as measured from the focal male´s position, was identical in two populations studied, although the mean inter-male distances differed significantly (Römer and Bailey 1986).

In the first paper of this series, the spacing of three populations of the katydid *Sphagniana sphagnorum* was studied in their bog habitat; their mean inter-male distances varied between 5.1 and 8.4 m (Morris et al. 2018). This species has an unusual frequency modulation in its calling song, as it continuously switches between two song modes in the high-audio and ultrasonic ranges (peaks at 17 and 34 kHz, respectively; Morris 1970). The authors asked whether males could use differential spectral attenuation to space themselves with conspecific rivals. As expected, the data of sound transmission experiments in one of the populations indicated, that each of these two song modes are differently affected by excess attenuation, so that their relative amplitudes are dominated by the HF mode at distances nearer than 6 m, whereas the audio mode dominates strongly at greater distances. The reported mean inter-male distances of 5.1, 6.4, and 8.4 m roughly approximates the distance at which the switch in the amplitudes of both modes occurs. The results of an analysis of the song rates of nearest neighbour males indicated that those males separated by more than 4 m had similar song rates. At shorter distances of 4 m or less, however, one male of the pair sang at a much higher rate than the other. The results of acoustic playbacks also indicated that the song rate is an important acoustic cue in male–male interactions (Morris et al. 2018). Altogether, these results suggest that males of *S. sphagnorum* space themselves in the population using acoustic cues and that when these cues indicate closer inter-male distances, aggressive acoustic interactions will occur.

However, so far, it is completely unknown how males estimate the distance to their neighbours, and how the calling songs as perceived at varying distances are represented in the afferent auditory pathway of male receivers. In this study, we investigated the representation of the *S. sphagnorum* song in receptors and interneurons in the prothoracic ganglion, and how the neural response to the two modes changes over distance. Such a study would be incomplete unless we considered the effects of a potential source of masking noise in the natural population, namely, the song of another katydid species (*Conocephalus fasciatus*), which sings at the same time and location as *S. sphagnorum*. We also describe how *S. sphagnorum* escapes strong masking using the two frequency modes.

## Materials and methods

### Animals

Males of *S. sphagnorum* were collected in Canada and sent to Graz by mail for further experiments. These individuals were kept in a terrarium filled with moss maintained at a temperature of 27 °C and exposed to a 12 h light/dark cycle. They were fed with fresh lettuce, oat flakes and fish food ad libitum.

### Acoustic stimulation

Digitized recordings of the calling song of a male were taken and used to create model songs for playbacks. The song had been recorded from an isolated male at a distance of 15 cm using a B&K 2204 sound level meter, a B&K ¼″ 4135 microphone and a digitizing board (Keithley Instruments PCIP AWFG, 12 bit, 200 kilosamples/s). The song of *S. sphagnorum* is a prolonged trill with a characteristic, regular change in two song modes that differ in pulse structure and frequency spectrum. One mode is dominated by high-audio frequencies (maximum at 17 kHz), and the ultrasonic mode has its maximum at 34 kHz (for further details, see Morris et al. 2018).

The software Cool Edit Pro 2.0 was used to edit the playback signals for neurophysiological experiments. The song sequences consisted of 15 identical alternations of the audio and HF modes, each with a duration of 260 ms. Six different model songs were created, reproducing the SPL of each song mode at a given distance (1, 3, 6, 9, 12, and 16 m). The exact values of the respective SPL for each mode were taken from sound recordings in the field at these distances (Morris et al. 2018). Broadcasting these model songs during the neurophysiological experiments allowed us to simulate the perceived conspecific signals at different communication distances between males. The audio mode exhibited SPLs of 71, 63, 57, 52, 50, and 48 dB SPL at these distances; in the HF mode, these values were 78, 68, 57, 49, 43, and 36 dB SPL, respectively.

To study the influence of biotic background noise produced by a sympatric katydid species, the conspecific song of *S. sphagnorum* was presented together with the song of *C. fasciatus*. The latter katydid communicates at the same time of day and in the same habitat as the former, with sound pulses repeated at a pulse period of 1.5 ms, covering a frequency that ranges from 30 to 80 kHz. For this reason, the *C. fasciatus* song may strongly mask the HF mode of *S. sphagnorum* song, whereas we assumed that the audio mode would be unaffected. *C. fasciatus* males broadcast a high intensity song (about 83–85 dB SPL at 50 cm). To simulate a situation in which a *Conocephalus* male song interferes with the perception of the conspecific signal at a distance between a male *S. sphagnorum* receiver and the masker of about 4 m, the sound SPL of the masking signal was constantly set at 57 dB SPL (by taking into account strong excess attenuation of the ultrasonic frequencies in the bog). In addition, to analyse the tuning properties of both receptors and interneurons, pure tone sound pulses (50 ms duration) were generated using Cool Edit software for sound frequencies ranging between 5 and 60 kHz at a sampling rate of 192 kHz. The threshold at a given frequency was determined as the SPL when the neuron responded with suprathreshold action potential activity in at least three out of the five stimulus presentations. In addition, sound pulses were presented at a constant SPL that ranged from 65 to 80 dB SPL to generate iso-intensity functions.

The software Cool Edit Pro 2.0 was used for playback in neurophysiological experiments. All signals were processed using an Edirol 101 audio interface at a sampling rate of 192 kHz. Signals were amplified with an SA1 stereo power amplifier (Tucker Davis Inc., Alachua, Fl, USA) and broadcast by two multi-field magnetic speakers (MF1) (Tucker Davis Inc., Alachua, Fl, USA). The speakers were positioned next to one another to one side of the longitudinal body axis of the preparation at a distance of 15 cm. Sound pressure levels at the preparation were calibrated at the position of the ears using a ½″ microphone (type 2540, Larson Davis, Depew, NY, USA) that was connected to a sound level meter (CEL 414, Casella, Bedford, UK).

### Intracellular recordings

Animals were anesthetized with chloroethyl, and the antennae, mid- and hind legs were removed. The animals were fixed with the ventral side up on a holder using dental wax. The head was slightly tilted backwards and fixed tightly on the holder, and the tarsi of the forelegs were attached to thin wires using wax. An incision was made between the abdomen and thorax to reduce haemolymph pressure and tissue movements produced by breathing. The prothoracic ganglion was exposed by removing the prosternum and submerged in insect saline (ionic composition in g/l: 8.6 NaCL, 0.74 KCL, 0.76 CaCl_2_, 2.38 HEPES). The ganglion was stabilized between a small metal platform on its dorsal side and a metal ring on its ventral side with the platform serving as a reference electrode for intracellular recordings. A Flaming/Brown micropipette puller (Model P-87, Sutter instruments co, Novato, CA, USA) was used to produce microelectrodes with a resistance of 70–100 MΩ (1 mm OD, 0.58 mm ID, science products, Hofheim, Germany). A Leitz micromanipulator (model M; Leica Microsystems) allowed us to approach the electrode with high precision. The depth of the microelectrodes was measured using a Mitutoyo absolute digimatic indicator (ID-C112X; Mitutoyo Corporation). Intracellular recordings were mainly performed in the anterior part of the prothoracic ganglion within or close to the auditory neuropil (Römer et al. 1988; Kostarakos and Römer 2015). Neuronal responses were amplified by a BA-01X amplifier (npi electronic) in the bridge mode.

### Terminology

The terminology of the neuron types was established based on their frequency selectivity. “LF” indicates low-frequency selectivity and was used to refer to neurons that responded more strongly to the audio component of the song, whereas “HF” is used for neurons that responded more strongly to the HF component of the song. The third letters indicate either receptors (R) or interneurons (I). We made no attempt to stain and characterize neurons morphologically by dye injection, because we had only a limited number of specimens available, but we tried to characterize the distance–response functions of as many neurons as possible. Nevertheless, it was possible to clearly distinguish between receptors and interneurons based on their EPSP or IPSP activity and by manipulating the response strength by current injection.

### Data recordings and analysis

All recording channels were digitized at a 20.8 kHz and 16-bit amplitude resolution with 0.153 mV per increment using a CED 1401 micro3 data acquisition interface. Data were recorded to the hard disk drive of a PC using Spike 2 software (Cambridge Electronic Design). Neural recordings were also displayed on an oscilloscope (5502; Hung Chang) and monitored using a TG1000M multimedia speaker (dynavox). Neural recordings were analysed off-line using Neurolab software (Knepper and Hedwig 1997). Neural responses were analysed using peri-stimulus time (PST) histograms and averages of the instantaneous spike frequency or postsynaptic membrane potentials.

## Results

To examine the neural basis of acoustic distance estimation in the context of spacing behaviour, we analysed the auditory receptors and interneurons and their responses to the audio and HF modes to model songs representing different male–male distances.

### Auditory receptors

**Fig. 1:**
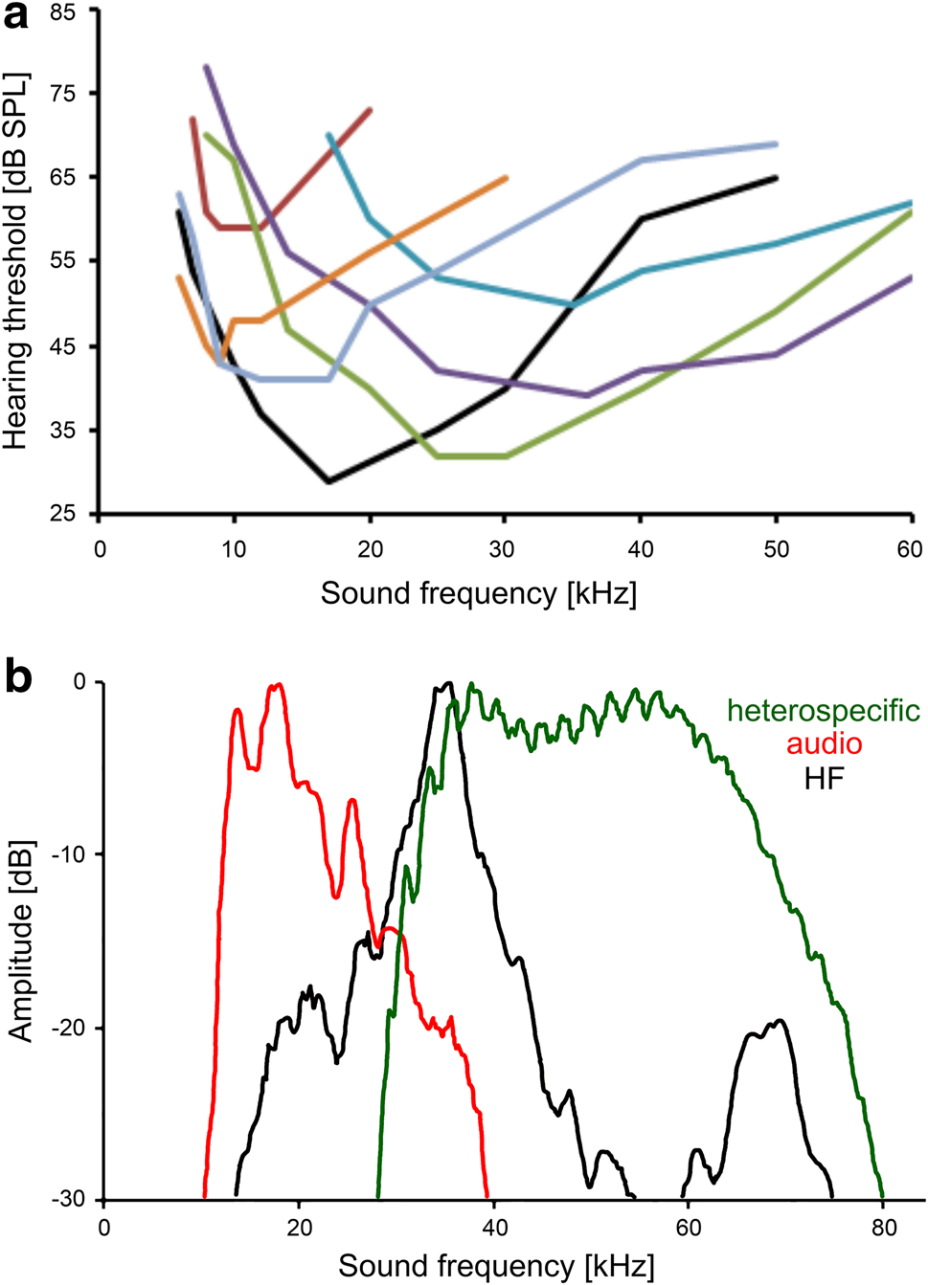
**a** Tuning properties of seven different auditory receptors recorded in one animal. **b** Spectrum of the audio (red) and HF mode (black) of the *S. sphagnorum* song, as well as of the heterospecific song of *C. fasciatus* (green)

Since the responses of the neurons to the audio and HF modes of the song strongly depended on their frequency sensitivity, we initially analysed their tuning. Figure 1a shows the tuning of seven auditory receptors, all recorded in one individual. They differ in their best frequency and absolute sensitivity, ranging from 9 to 36 kHz and 30 to 60 dB SPL, respectively. Best frequencies of all 21 receptors studied fell within a range of 7–45 kHz; no receptor was identified that was tuned below 7 kHz. The comparison with the spectral content of the audio and HF modes of the song (Fig. 1b) would suggest that the tuning and sensitivity of these receptors strongly determine their responses to both song modes at different distances.

**Fig. 2:**
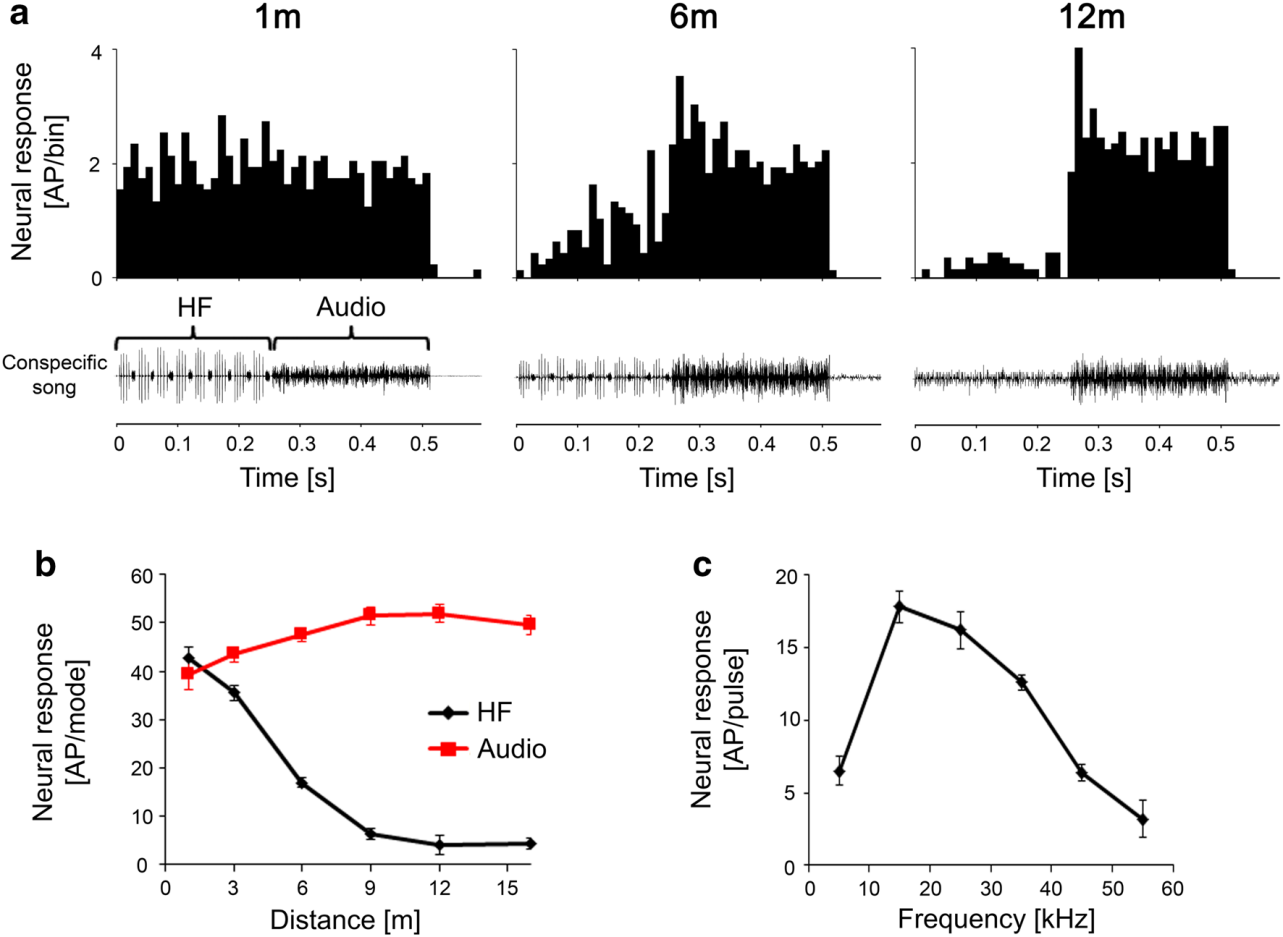
PSTHs of responses of an LF receptor to stimuli representing distances of 1, 6 and 12 m from a singing male **(a**) The neuron is tuned to 15 kHz as shown by the iso-intensity function in **c** (sound intensity = 65 dB SPL). The distance–response function to the audio and HF mode of the song is shown in **(b**) In this and all the following distance–response functions, the responses to the audio mode are shown in red, and those to the HF mode, in black. Note that the response to both modes is the same at 1 m due to the high sensitivity of this receptor

For example, Fig. 2a shows the responses of a receptor to both song modes at communication distances of 1, 6, and 12 m as PST histograms. The iso-intensity function (Fig. 2c) revealed a tuning to 15 kHz; the receptor had a low threshold that approached 30 dB SPL. Due to both the tuning and higher SPL of the audio mode at greater distances, the receptor responded exclusively to the audio mode at distances that exceeded 12 m (Fig. 2b, c). As the distances decreased, the energy of the HF mode increased, resulting in an increase in the response to the HF mode, and the distance response function and PSTHs revealed similar responses to both modes at a distance of 1 m. The results of an analysis of less sensitive LF receptors revealed a distance–response function with exclusive responses to the audio mode beginning at distances of 12–15 m, which increased at shorter distances, whereas the response of these receptors to the HF mode had a threshold distance of 3–6 m.

**Fig. 3:**
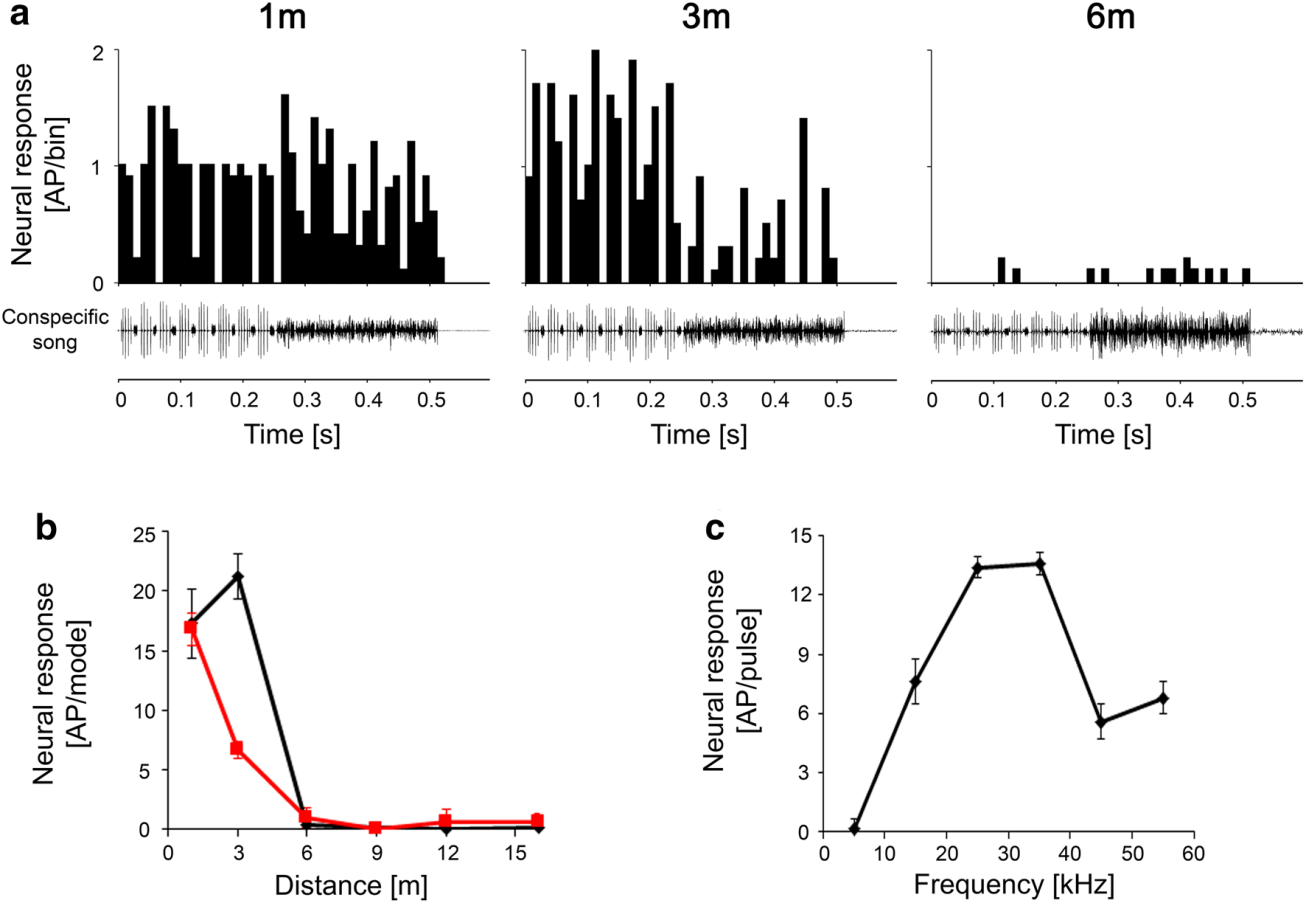
PSTHs of responses of a HF receptor to stimuli at distances of 1, 3 and 6 m from a singing male (**a**). The neuron is tuned to 30 kHz (**c**), sound intensity = 80 dB SPL. The distance–response function to the audio and HF mode of the song is shown in **b**. Note that there is no response at 6 m due to the high threshold of this receptor

Another auditory receptor was tuned to higher frequencies that ranged from 25 to 35 kHz (Fig. 3c). The PST histograms of responses at distances of 1, 3, and 6 m, as well as the distance response function, show that this HF receptor was less sensitive. Therefore, it began to respond preferentially to the HF mode of the song at a distance of 3 m, whereas the response at 1 m was identical for both modes (Fig. 3a, b).

### Responses of interneurons

These results indicate that auditory receptors respond differently to the two song components depending on their tuning and overall sensitivity. We classified auditory interneurons into three basic categories based on their responses to the song components. ‘Low-frequency interneurons’ (LFINs) are tuned to lower frequencies and responded preferentially to the audio mode of the song, especially at larger communication distances. ‘High-frequency interneurons’ (HFINs) are tuned to ultrasonic frequencies and responded much more strongly to the HF component of the song at close range. ‘Switch interneurons’ (Switch INs) were sensitive to both spectral components of the song, but exhibited a typical activity switch at a distance of 3–6 m.

#### Low-frequency interneurons (LFINs)

**Fig. 4:**
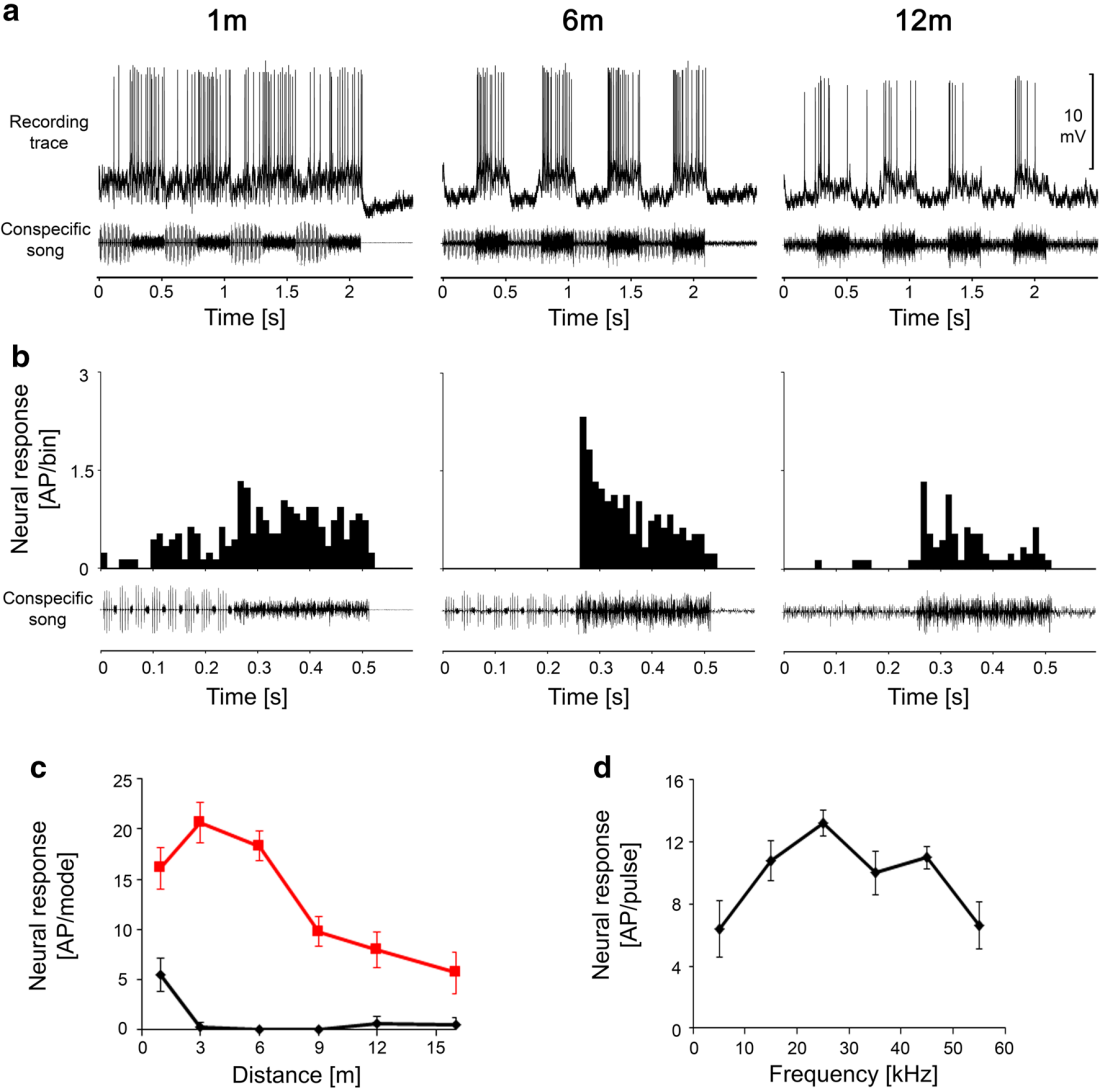
Intracellular recording traces (**a**) and PSTHs of responses (**b**) of an LFIN to stimuli at distances of 1, 6, and 12 m from a singing male. Note the nearly exclusive responses to the audio mode (**a**). **c** Distance–response function to the audio and HF mode of the song. **d** Iso-intensity function at 80 dB SPL

Figure 4a shows recording traces of an LF Interneuron at communication distances of 1, 6, and 12 m. This neuron responded almost exclusively to the audio component at all communication distances tested; a minor amount of activity in response to the HF song mode was observed only at a distance of 1 m. The distance response function (Fig. 4c) showed an increasing magnitude of response to the audio mode as the distance decreased. Other LFINs, which were more sensitive, were strongly and exclusively excited by the audio mode at great distances of 15 m and more. Whereas the response to the audio mode remained almost identical at all distances, the response to the HF mode increased as the distance decreased (Fig. 7a).

#### High-frequency interneurons (HFINs)

**Fig. 5:**
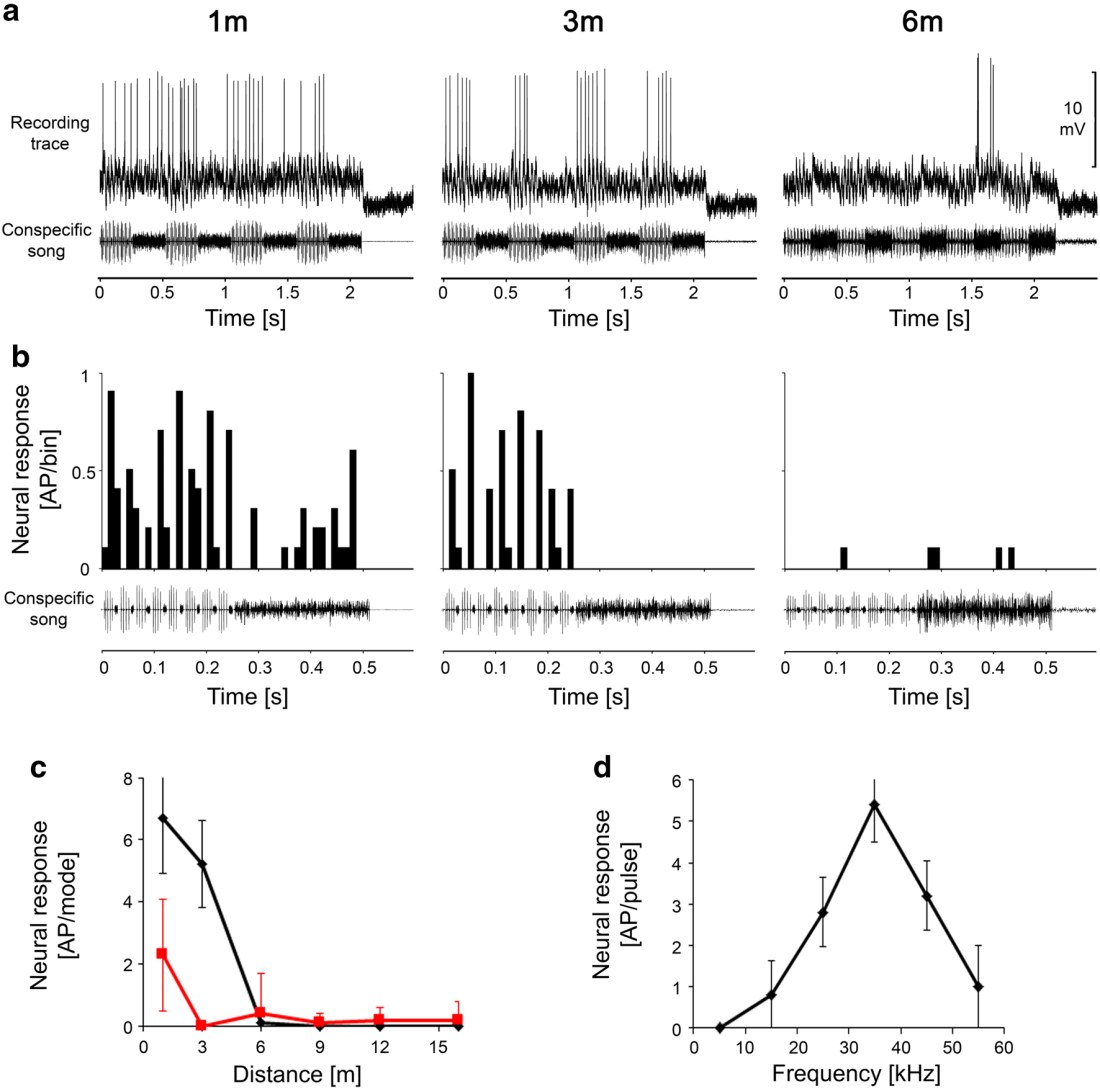
Intracellular recording traces (**a**) and PSTHs of responses (**b**) of a HFIN to stimuli at distances of 1, 3, and 6 m from a singing male. The neuron is tuned to 35 kHz (**d**) (sound intensity = 65 dB SPL). Note the high threshold of the neuron and nearly exclusive response to the HF mode in **c**

Figure 5a illustrates recording traces of a HF interneuron at communication distances of 1, 3, and 6 m. The neuron was selectively tuned to 35 kHz (i.e., the spectrum of the HF mode of the song; Fig. 5d). However, because the sensitivity was rather low, almost no response was elicited at a distance of 6 m. Between 6 and 1 m, the response strongly increased, and at 3 m, the neuron responded exclusively to the HF mode, with activity triggered by each of the eight series of HF pulses (Fig. 5b). As a general feature of these HFINs, we identified their relatively high threshold, so that their distance response functions showed no responses at all at distances greater than 6 m, but increased sharply at shorter distances.

#### Switch interneurons

**Fig. 6:**
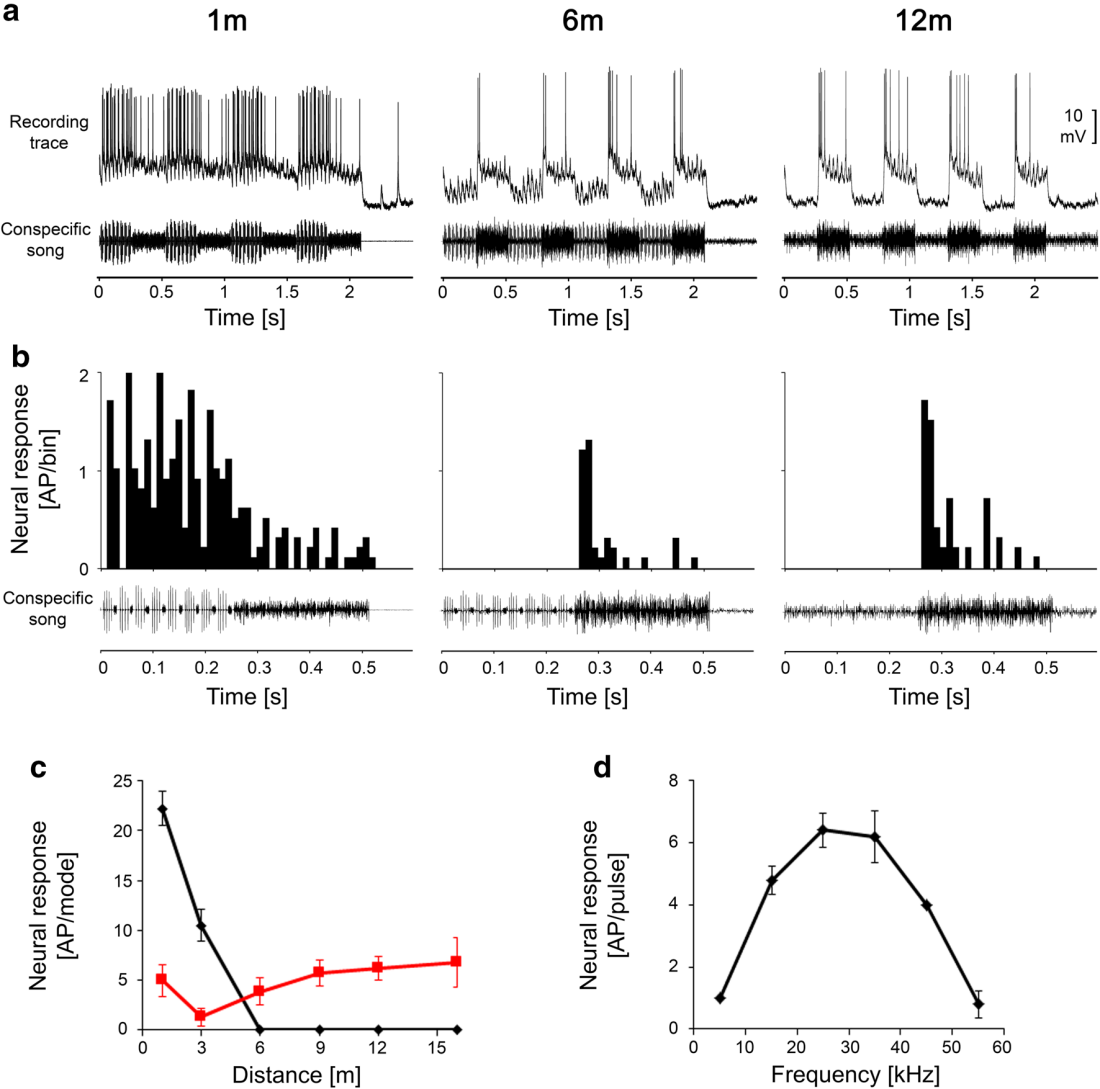
Intracellular recording traces (**a**) and PSTHs of responses (**b**) of a switch IN to stimuli at distances of 1, 6, and 12 m from a singing male. The neuron is tuned to frequencies that include both song modes (**d**) (sound intensity = 65 dB SPL). The neuron responded exclusively to the audio mode at distances between 6 and 12 m (see subthreshold EPSP and spike activity in **a**), but at close range, it switched its activity to the HF mode (**c**)

Whereas the two previously described classes of INs could be characterized by the fact that they were selectively tuned to one of the two modes of the song, the switch INs were rather broad-band interneurons that were tuned to the intermediate frequencies of the two song components. These neurons displayed a typical switch in their activity by responding stronger or exclusively to the audio mode at distances of 6 m or greater but responding selectively to the HF mode of song at close range. All neurons in this class displayed this ‘switching’ behaviour at a distance between 3 and 6 m. Figure 6a shows recording traces of one such switch IN at distances of 1, 6, and 12 m; the neuron was tuned to 25–35 kHz with a rather characteristic broad-band frequency. A conspecific song played at a distance of 12 m elicited strong EPSP activity in this neuron, but exclusively in the audio mode, and we observed AP activity mainly at the onset of the audio mode (see also PSTH in Fig. 6b). The same was true at a distance of 6 m, but subthreshold EPSPs were also elicited by the HF mode of the song. However, the response was entirely reversed at the close range of 1 m, at which the cell then responded strongly to the HF mode, whereas the response to the audio mode was reduced. The distance response function shown in Fig. 6c demonstrates that the switch in activity occurs at a distance between 3 and 6 m.

**Fig. 7:**
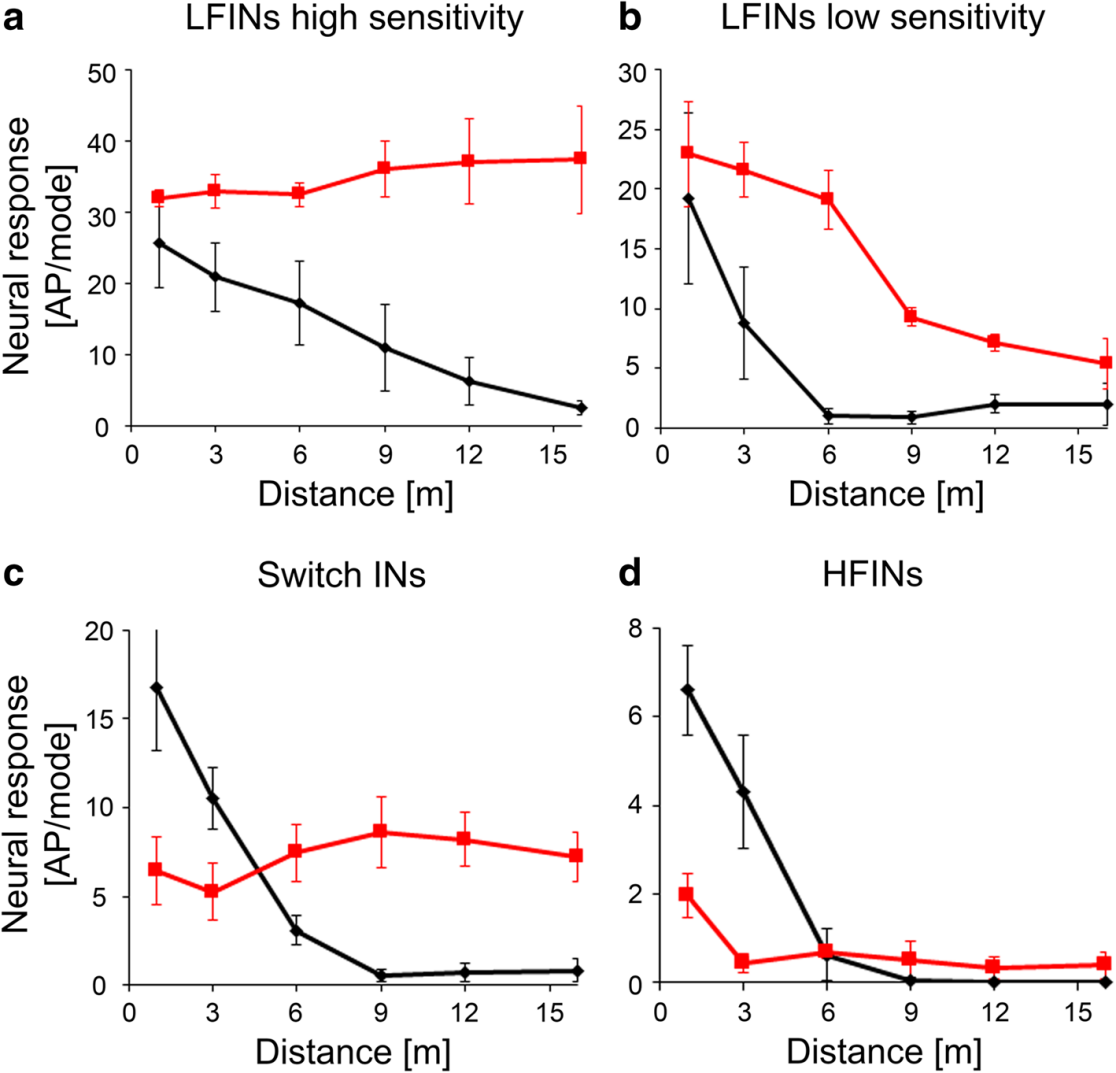
Summary of distance–response functions of all types of interneurons, including LFINs with high (**a**) and low sensitivity (**b**), switch INs (**c**), and HFINs (**d**) (*N* = 5, 3, 8, and 3, respectively)

An overview of the distance–response functions for all interneurons is given in Fig. 7, in which we differentiated between LFINs with high and low sensitivity (Fig. 7a, b). The response to the audio mode remained high and almost unchanged over all distances in those interneurons with high sensitivity, whereas the response to the HF mode increased linearly as the distance decreased. A linear increase of responses to the audio mode was observed at shorter distances in less sensitive LFINs, and the response to the HF mode increased strongly at distances less than 6 m. The latter was also true for HFINs (Fig. 7d), which displayed a moderate response to the audio mode only at a distance of 1 m. In contrast, the switch INs (Fig. 7c) displayed a constant level of response to the audio mode at all distances, but the strength of activity between both modes was reversed at shorter distances (i.e., less than 6 m) due to a strong increase in the response to the HF mode.

### Masking of responses due to acoustic background of *C. fasciatus*

**Fig. 8:**
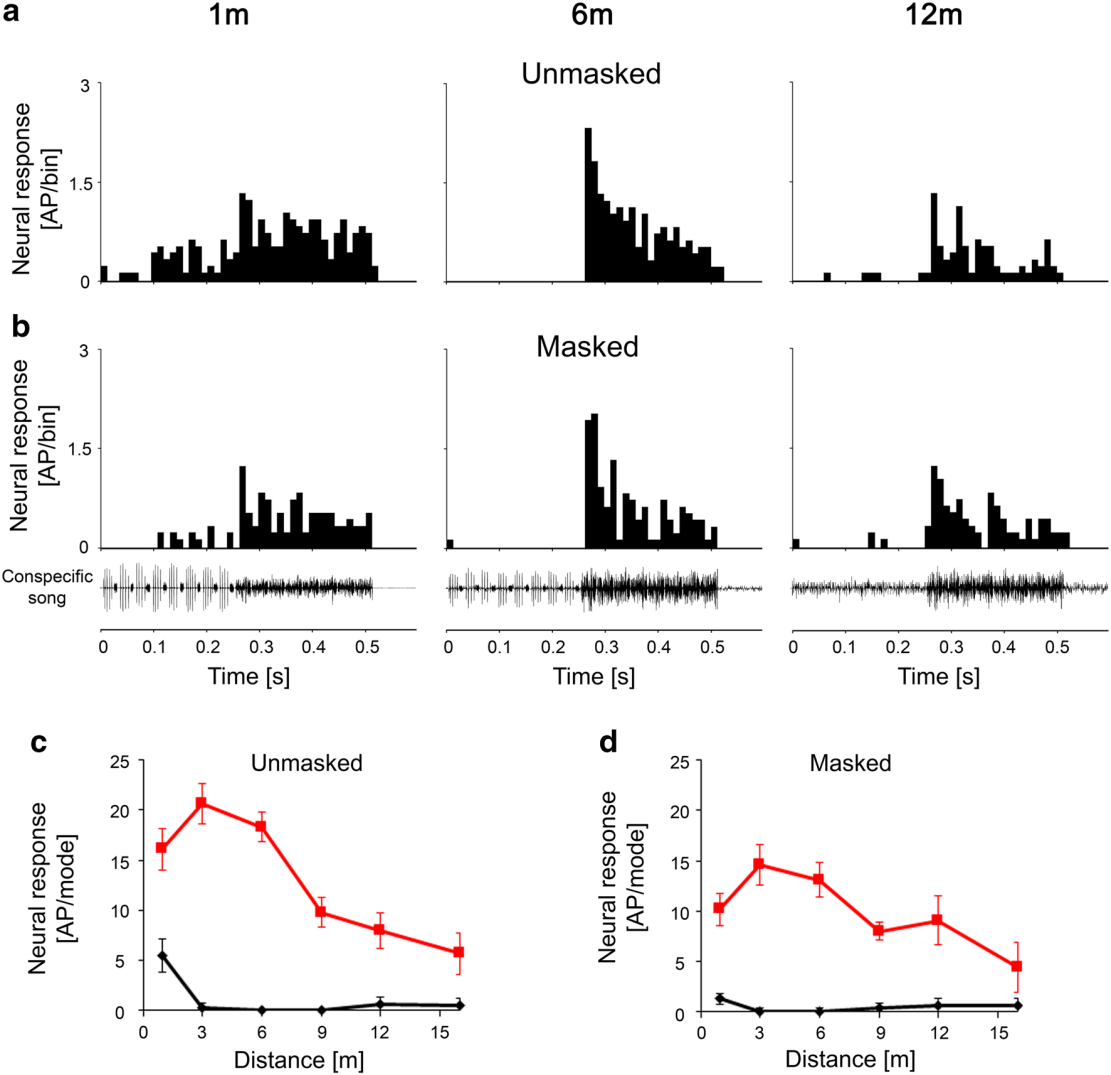
Comparison of responses of a less sensitive LFIN in the unmasked condition (**a, c**) and under conditions of continuous masking by *C. fasciatus* song (**b, d**). Note that the selective response to the audio mode is still preserved, despite the effects of masking, at all distances

**Fig. 9:**
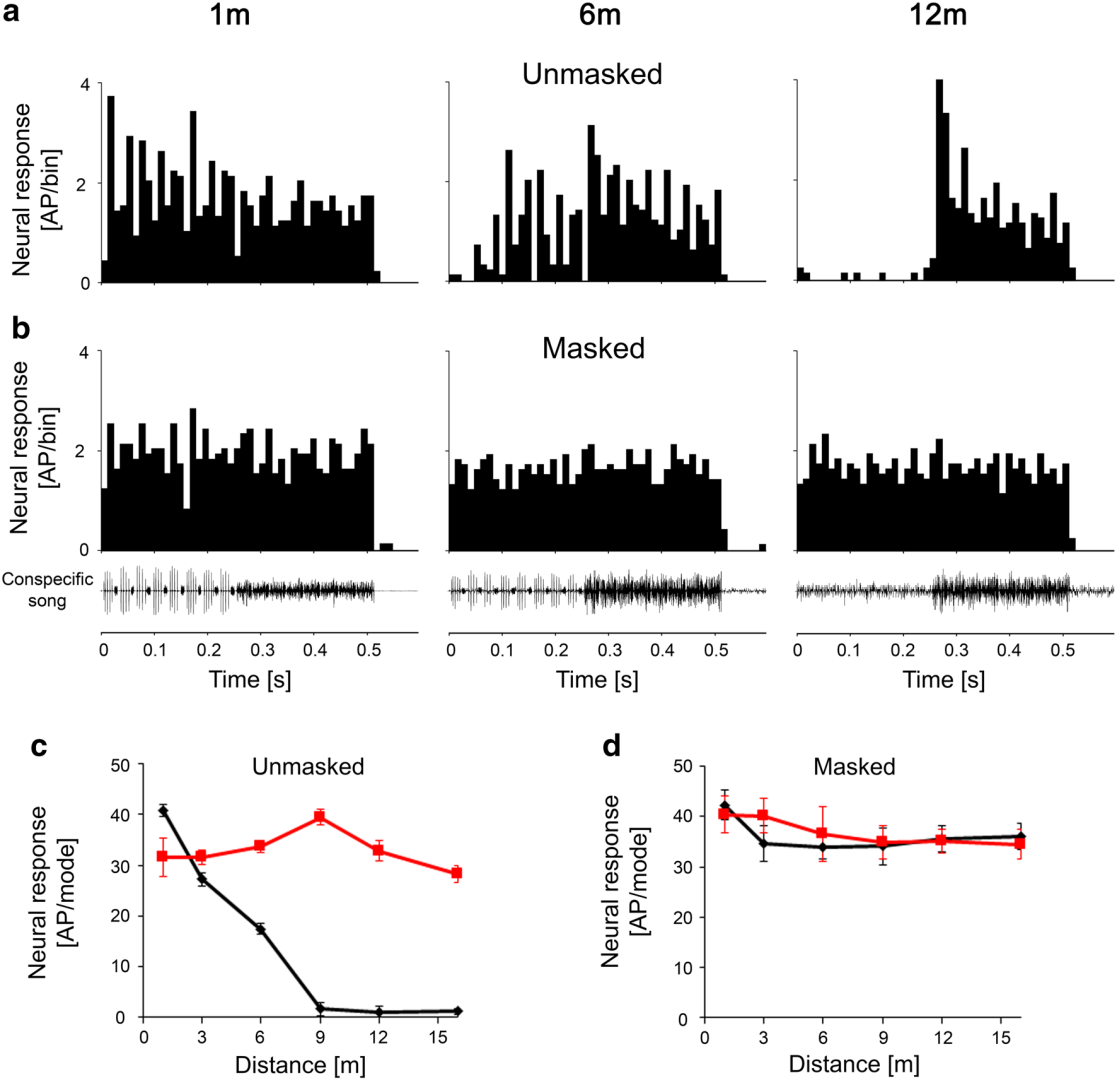
Comparison of responses of a sensitive LFIN in the unmasked condition (**a, c**) and under the continuous masking condition with *C. fasciatus* song (**b, d**). Note the full effects of masking on the selective response to the audio mode, even at greater distances

**Fig. 10:**
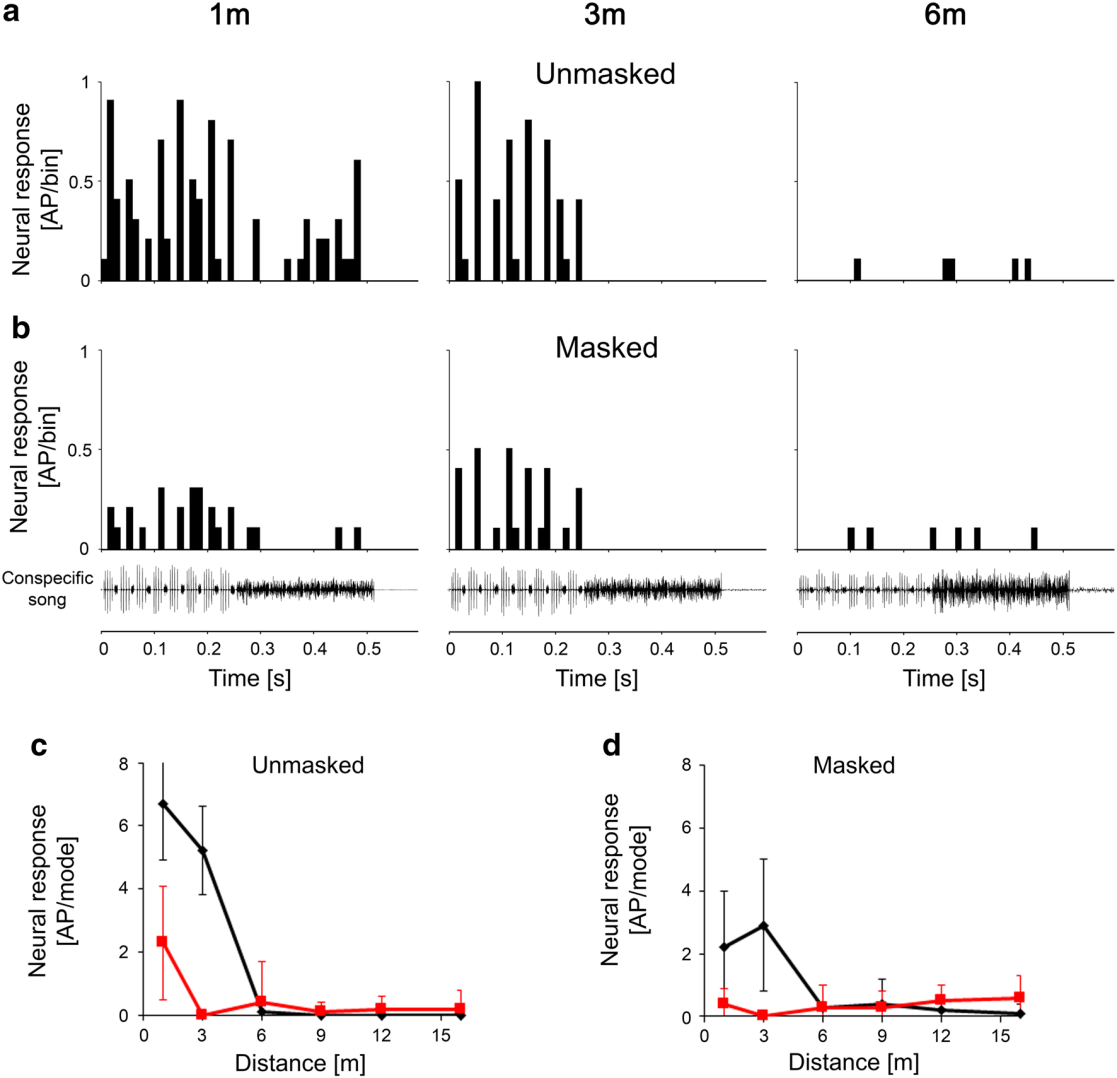
Comparison of responses of an HFIN in the unmasked condition (**a, c**) and under conditions of continuous masking by *C. fasciatus* song (**b, d**). Note that the selective response to the HF mode was still preserved despite the effects of masking, but the response strength to the HF mode was reduced

The sympatric katydid *C. fasciatus* produces an ongoing song in the same habitat as *S. sphagnorum* at frequencies of about 30 kHz and higher, which can potentially mask the response of *S. sphagnorum* to the HF mode. Figure 8 illustrates that a less sensitive LFIN was almost unaffected by this background noise. It responded exclusively to the audio mode at all distances, and the distance response function indicated only a moderate decrease in the level of excitation (Fig. 8c, d; data from one individual; 2 further LFINs showed similar responses to the masker). These results differed from those of sensitive LFINs, where the exclusive response to the audio mode at larger distances was completely masked at all distances by the song of *C. fasciatus* (Fig. 9a, b). This was also evident when examining the distance response function for both components; the response to the HF mode was identical in strength at all distances to that of the response to the audio mode (Fig. 9c, d; data shown are from one individual; *N* = 3 for sensitive LFINs, *N* = 5 for switch INs; *N* = 2 for HFINs). The example of an HFIN in Fig. 10 demonstrates that the ultrasonic masker did not abolish the exclusive response to the HF mode of the song, but rather reduced the response to the HF mode at close range. These different sensitivities to the masking signal of *C. fasciatus* in the various interneurons were also confirmed in experiments with receptors, differing in tuning and absolute sensitivity (*N* = 15).

## Discussion

When female katydids make phonotactic decisions between several mates in the field, or males space out in the population and maintain a given distance to their neighbours, they need to estimate the distance to a sound source. The ranging hypothesis that has been proposed by various authors (McGregor and Krebs 1984; Morton 1986; Naguib and Wiley 2001; Morris et al. 2016) suggests that receivers use frequency-dependent excess attenuation as a sensory cue to estimate their distance from a signaller. This is particularly interesting with respect to S. sphagnorum, since high and low frequencies are separated as a frequency modulation in time between the regularly changing HF and audio modes in this insect. Katydids could use such spectral information provided by the series of frequency analyzers in the crista acustica (Oldfield 1983; Stumpner 1996; Stölting and Stumpner 1998; Hildebrandt 2014). The aim of the present study was to identify neuronal correlates in the afferent auditory pathway that were indicative of the spacing behaviour displayed by male *S. sphagnorum* in the field (Morris et al. 2018). In the three studied populations, males maintained average distances between 5.1 and 8.4 m from each other, and their song interactions indicated that they sing with a similar song rate at these and greater distances. However, at shorter inter-male distances, the song rates between two males differed significantly, indicating that agonistic acoustic interactions take place when one male approaches the other too closely. With this ecological background, our neurophysiological approach was taken to find out (1) how critical information about the rate of mode changes is represented in the array of auditory receptors and interneurons and (2) what happens in the representation of the conspecific song at distances less than 6 m away from a signaller, where behavioural changes have been observed to occur. Furthermore, (3) analyses were conducted to study the masking effects that occur when the sympatric katydid *C. fasciatus* sings.

One important way to study the functional organization of the afferent auditory pathway is to investigate the responses of sensory afferents and interneurons to acoustic stimuli that mimic sound signals that occur in nature (Suga 1984; Römer 1987). In the context of distance estimation, it is necessary to know how the transmission channel affects the signal in the frequency and time domains to correctly simulate communication distances in neurophysiological experiments. In our study, the song models were created based on an analysis of the male *S. sphagnorum* song and how it changes due to frequency-dependent filtering in the natural habitat, which affects the audio and HF modes differently (Morris et al. 2018). The model songs created thereby represented realistic amplitudes of the two song modes for each distance.

### Auditory receptors

When auditory receptors differ in their sensitivity and/or tuning, they can provide the sensory basis for distance coding, since each receptor will respond at a different threshold distance. Whereas these receptors are usually found in the same hearing organ, a striking case for range fractionation has been reported in bladder grasshoppers (Orthoptera, Pneumoridae), which have six pairs of serially repeated abdominal ears derived from proprioceptive pleural chordotonal organs (van Staaden and Römer 1998; van Staaden et al. 2003). Due to the different absolute sensitivity of each ear, the threshold distance at which receptors start to respond varies from about 10–100 m (van Staaden et al. 2003). In *S. sphagnorum*, the responses of auditory receptors to the conspecific song at various distances could be predicted from their frequency tuning, absolute sensitivities, and the relative amplitudes of both modes at a given distance. The best frequencies for all 21 receptors for which we obtained tuning curves ranged from 7 to 45 kHz and covered the main frequency range of both song modes. The receptors that were tuned to the audio mode were the most sensitive (Fig. 1), and consequently, they responded strongly and exclusively to the audio mode at both greater and intermediate distances, but the responses were non-selective at close range. We did not test the responses of these receptors for distances greater than 16 m, but their threshold of 30 dB SPL at 17 kHz and the amplitude of 45–50 dB SPL for the audio mode at 18 m in the bog habitat (Morris et al. 2018) suggest that these highly sensitive receptors would respond well at distances exceeding 30 m. Similarly tuned but less sensitive receptors responded exclusively to the audio mode except at very short distances. In contrast, receptors tuned to ultrasonic frequencies did not respond at all at distances greater than 6 m due to their reduced sensitivity, and the HF mode of the song attenuated more rapidly over greater distances. When a similar approach was taken with the Australian katydid *M. marki*, comparable results were obtained since the individual receptors had different tuning and absolute sensitivities. Each receptor differed in terms of the distance at which it began to respond (threshold distance), which resulted in a range fractionation in the coding of the distance from a signaller (Römer 1987). Unlike *S. sphagnorum*, however, all syllables of the song were affected by the same frequency filtering by the transmission channel in *M. marki*, so that the rate of the syllable was represented in the afferent discharges as soon the overall SPL reached suprathreshold levels (Römer and Bailey 1986). Moreover, the tonotopic arrangement of the axonal endings of these receptors combined with their range fractionation resulted in a specific spatial distribution of afferent activity in the auditory neuropile. Pollack and Imaizumi (1999) interpreted this as an “odotopic map”, a systematic relationship between the distance from the sound source and the spatial distribution of activity in the auditory neuropile. Given that tonotopicity is a general feature that has been observed in the ear and the auditory neuropile of all katydids studied thus far (Oldfield 1983; Römer 1983; Stumpner 1996; Stölting and Stumpner 1998; Hildebrandt 2014), the signal of a distant singing *S. sphagnorum* male would be represented in the ventral part of the neuropile by rhythmic bursts of activity produced only in response to the audio part of song. Because of the additional input at intermediate distances of about 6 m being suprathreshold for HF receptors (which branch in the more dorsal part of the neuropile), alternating bursting activity is expected in both dorsal and ventral parts due to the alternating audio and HF song modes. Even if the most sensitive LF receptors would respond to both components of the song, the change in song modes would still be represented as rhythmic bursting activity in the dorsal neuropile. Thus, the population of receptors guarantees that important information about the song rate is fully represented in the auditory afferents at all communication distances.

### Interneurons

The responses of interneurons to the conspecific song at various communication distances differed strongly, again depending on their tuning and absolute sensitivity, but also on intrinsic properties and excitatory and inhibitory synaptic interactions. At distances of 6 m and more, LFINs showed highly selective responses to the audio mode, so that the information about the mode rate is encoded in this group of interneurons. As neural correlates for the spacing of males in the field, the two other groups of interneurons appear to be of special interest. HFINs have high thresholds and, therefore, begin to respond to the HF mode at distances shorter than 6 m. At similarly short distances, the switch INs exhibited a strong shift in their response to both modes, responding to the audio mode at larger distances and the HF mode at shorter distances. The shift happened with song parameters representing distances of 3–6 m. Although these results only represent a correlation between the observed spacing and song interaction in the field, it is tempting to speculate that the activity of these two groups of interneurons transmits specific information to the brain of receivers about inter-male distances: that they are closer to a singing neighbour than can be tolerated, and that they should initiate aggressive song interactions at distances shorter than 4 m (Morris et al. 2018).

**Fig. 11:**
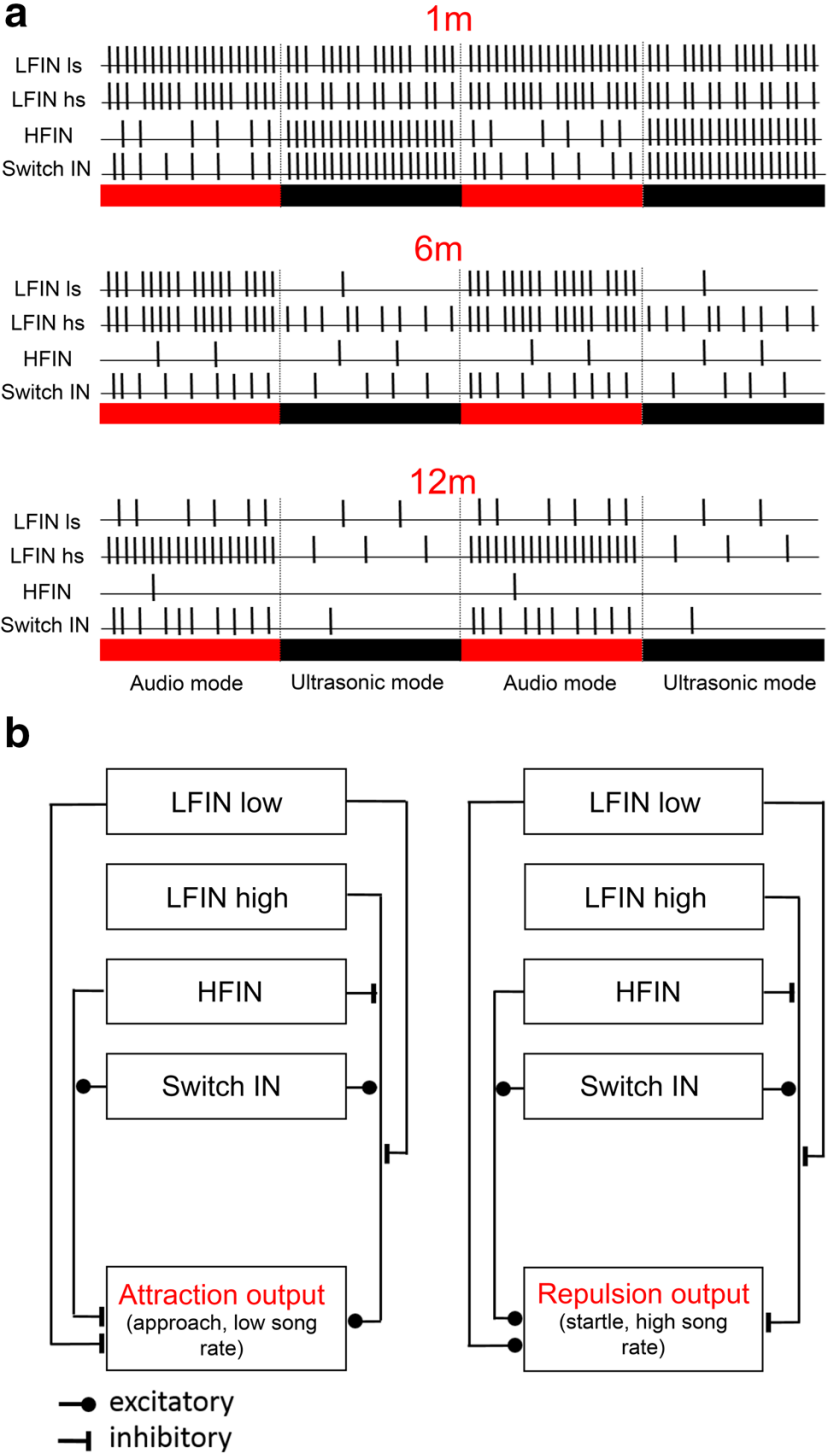
**a** Schematic presentation of the simultaneous action potential activity of four INs in response to the two song modes at different distances. The response strength is shown on a relative scale, for each IN (100% activity = 20 APs, 5% activity = 1 AP). **b** Hypothetical model of neural connectivity in neural networks of the brain and their possible influence on the output for two specific behaviours (“attraction” or “repulsion”). Note that the pathway for a specific output can also involve intermediate neurons, for example those mediating inhibition

How could a hypothetical network in the brain of a receiver reliably extract the information about distance to a rival male? Figure 11a illustrates schematically the simultaneous activity of four described interneurons in response to the frequency-modulated song at close range, as well at intermediate and large distances. A hypothetical model of signal processing in the brain is presented in Fig. 11b to demonstrate how the combined activity of these interneurons can elicit spacing behaviour via attraction to a distant chorus and repulsion from nearby rivals. The premise of the model is that HFINs and less sensitive LFINs (LFIN ls) drive the “repulsion” output that results in agonistic song interactions and/or movement away from a rival (repulsion) at distances shorter than 6 m. By contrast, the activity of the sensitive LFINs (LFIN hs) activates the “attraction” output with song parameters representing distances greater than 6 m. The switch INs with their response to the audio mode at greater distances, and to the HF mode at shorter distances, support both pathways, depending on their co-activation with other INs. A co-activation of a HFIN, a less sensitive LFIN with a switch IN in response to the HF mode would be indicative of a distance shorter than the ‘tolerable’ inter-male distance. Since the less sensitive LFIN inhibits the excitatory pathway of the sensitive LFIN; however, the “attraction” pathway cannot be activated by the audio mode at short distances. In contrast, co-activation of a switch IN and a sensitive LFIN in response to the audio mode would provide information about a distance exceeding the inter-male distance, which could then activate the “attraction” pathway. Since the response of the other two INs is low at greater distances (compare with Fig. 11a), this pathway will be released from inhibition.

The simultaneous combination of activity will also lead to different degrees of output activation, depending on the different degree of activation at different communication distances. This could provide reliable information about the distance to a neighbour. However, even the activity of single INs could provide a rough estimate about communication distances, based on the differential activity in response to the audio compared to the ultrasonic mode. For example, the response difference of the sensitive LFIN is very high at large distances, but the difference decreases with decreasing distances (Fig. 11a).

In a similar way, the summarized responses in Fig. 11a also demonstrate that at any distance from a rival male, the important information about the song rate, as expressed in continuous changes of high and low activity, is represented in the combined activity of interneurons. At large distances, for example, the song rate is well represented by the sensitive LFIN, at short distances by the switch IN and the HFIN. Even under the strong masking conditions of the sympatric species, this information is provided by the less sensitive LFIN.

### The two song modes and release from masking

In contrast to species-rich habitats like tropical rainforests with many species of acoustic insects creating a high level of background noise (Ellinger and Hödl 2003; Lang et al. 2005; Diwakar and Balakrishnan 2006), the only other insect singing at the same time and location as *S. sphagnorum* is the conocephaline katydid *C. fasciatus*. Accordingly, the masking effects should be less severe. The spectrum of *C. fasciatus* song completely overlaps the HF mode of *S. sphagnorum* (Fig. 1b); thus, we expected an escape from masking due to the frequency separation of the signal and masker in the audio mode, as was found in another katydid (Siegert et al. 2013; Kostarakos and Römer 2015). The degree of masking varied in different groups of interneurons, and was surprisingly more severe for some LFINs than HFINs (compare Figs. 9, 10). This is because some LFINs are highly sensitive and respond strongly even to the moderate broadcast level of the masker, resulting in complete masking of the selective response to the audio mode at both larger and intermediate distances. Less sensitive LFINs and HFINs (Figs. 8, 10) still provided selective responses to either the audio or HF mode under the same masking conditions, as the responses to the masking signal at a distance of 6 m were extremely low.

These neurophysiological data corroborate findings from field studies in which the distribution of both katydid species in one of the populations was examined (Morris et al. 2018). In one part of the population, *C. fasciatus* singers were densely distributed among interspersed *S. sphagnorum* singers, whereas *C. fasciatus* males were not observed in the nearby area, where only *S. sphagnorum* males were singing. Still, the spacing of *S. sphagnorum* did not differ in both areas, and the results of a statistical analysis of nearest neighbour distances showed that the potential masking species had no impact on the spacing of *S. sphagnorum*. We hypothesize that the properties of the auditory receptors and interneurons, combined with the separation of two spectra in two different song modes, enable *S. sphagnorum* to escape the effects of masking interference produced by *C. fasciatus*. This combination would represent yet another mechanism that receivers use to cope with biotic noise (Römer 2014).

However, we should consider that the masking problem for male and female *S. sphagnorum* may be different. Most males sing from some higher elevated positions, whereas the flightless females must approach conspecific males on the bog surface, where males of *C. fasciatus* broadcast their high amplitude song (about 83–85 dB SPL at a distance of 50 cm). Thus, females are exposed on average to higher masking levels than those used in our study. Little is known about phonotactic approaches of females, but when field matings have been observed, all occurred in trees. Furthermore, lone females without males are frequently seen in short trees, giving the impression that they may climb up from the bog to listen for male calls. Here, they may either escape the high masking levels of *C. fasciatus*, or alternatively, they use such elevated positions to better localize males (Rheinlaender and Römer 1986).

## Abbreviations

AP: Action potential
IN: Interneuron
LFIN: Low-frequency interneuron
HFIN: High-frequency interneuron
HF: High frequency
Ls: Low sensitivity
Hs: High sensitivity
